# A Statistical Modelling and Machine Learning Approach for Textile Wastewater Treatment: Response Surface Methodology, Random Forest Regression and Monte Carlo Analysis

**DOI:** 10.3390/membranes16070231

**Published:** 2026-07-02

**Authors:** Hafida Ayyoub, Sihame Barahi, Abderrahim Jbel, Mustapha Tahaikt, Mohamed Taky

**Affiliations:** Laboratory of Advanced Materials and Process Engineering, Faculty of Sciences, Ibn Tofail University, Kenitra P.O. Box 1246, Morocco; sihame.barahi@uit.ac.ma (S.B.); abderrahim.jbel@uit.ac.ma (A.J.); mustapha.tahaikt@uit.ac.ma (M.T.)

**Keywords:** textile wastewater treatment, aerobic ceramic membrane bioreactor, response surface methodology, Monte Carlo simulation, machine learning algorithm, random forest-Monte Carlo algorithm

## Abstract

Aerobic ceramic membrane bioreactors (AeCeMBR) have shown great potential in treating wastewater (WW) from the textile industry; however, their operation faces challenges such as process variability, membrane contamination, and the need for accurate prediction of treated water quality under varying conditions. In this study, chemical oxygen demand (COD) and turbidity were selected as key indicators, as they directly reflect organic load removal and solids separation efficiency in MBR systems. The effect of four operational parameters: hydraulic retention time (HRT), organic loading rate (OLR), mixed liquor suspended solids (MLSS), and transmembrane pressure (TMP), was investigated using a response surface methodology (RSM) based on a Box–Behnken design. A random forest (RF) model coupled with Monte Carlo simulation (MC) was also developed using 174 experimental data points to enhance predictive power and quantify uncertainty. The RSM model showed strong agreement with experimental results (coefficient of determination (R^2^) > 0.95), achieving approximately 96% removal for both COD and turbidity, with validation errors of less than 2%. MC simulation (10,000 iterations) was applied to assess the effect of ±10% variance under operating conditions, providing a probabilistic view of system performance. The RF-MC framework demonstrated high predictive accuracy, with strong correlations between predicted and observed values (R^2^ = 0.92 for COD and 0.97 for turbidity) and low uncertainty. Overall, this study proposes an integrated RSM, RF–MC approach for AeCeMBR systems, providing a robust and uncertainty-aware framework for process optimization and performance prediction under changing operating conditions.

## 1. Introduction

In line with the United Nations’ Sustainable Development Goals (SDGs), a shift to a more environmentally sustainable textile sector is required, particularly given that the industry currently discharges highly contaminated wastewater (WW) containing dyes, surfactants, salts, heavy metals, and heat-resistant organic pollutants [1,2]. According to the United Nations Environment Programme (UNEP), textile waste accounts for approximately 20% of global industrial water pollution and it is estimated that 50–400 L of WW are generated for each 1 kg of product [3,4]. This WW contains various contaminants and is characterized by high levels of pollution indicators such as total suspended solids (TSS), color, toxicity, and turbidity, as well as biochemical oxygen demand (BOD) and chemical oxygen demand (COD) [5]. The presence of high concentrations of suspended solids (SS) and water discoloration in the affected water bodies indicates long-term bioaccumulation of toxic substances [6]. Conventional WW treatment processes, such as coagulation, flocculation, activated sludge, and adsorption, are often inefficient when dealing with non-biodegradable organic matter and difficult dyes [7,8], resulting in non-compliance with stringent regulatory requirements and the production of large amounts of secondary sludge. Textile dyes present activated sludge with challenges of low biodegradability and recalcitrant properties. As a result, membrane bioreactor (MBR) technologies have emerged as highly promising alternatives [9]. MBRs combine biological degradation with a physical membrane barrier to produce high-quality effluent suitable for reuse, often achieving COD removal efficiencies of up to 95% and turbidity reductions of over 98% [5]. Among the variants, ceramic MBRs are particularly notable due to their superior mechanical and chemical stability compared to polymeric membranes, enabling effective ultrafiltration (UF) of complex effluents [10]. Nevertheless, the widespread adoption of these systems is significantly hindered by operational challenges, primarily membrane fouling and the resulting need for high transmembrane pressure (TMP), which contributes to energy-intensive operation [11]. Optimizing parameters such as hydraulic retention time (HRT), organic loading rate (OLR), and mixed liquor suspended solids (MLSS) is essential as it improves treatment effectiveness and reduces the high operational costs of MBR systems [12,13,14].

Conventional optimization methods based on one-variable-at-a-time (OVAT) tests are often ineffective and fail to take into account the interactive effects of variables, which frequently leads to suboptimal results [15]. A systematic method for modeling quadratic relationships and interactions between many factors is provided by response surface methodology (RSM), especially when combined with Box–Behnken design (BBD) [16]. This allows the optimal operating conditions to be identified with fewer experiments [17]. WW treatment is one area in which RSM has been successfully applied to optimize pH, dosage and contact time in adsorption processes, yielding models with R^2^ > 0.95. Nevertheless, RSM presupposes underlying quadratic relationships and may fail to take into account higher-order complexities or multicollinearities in datasets [18]. Complementing these methods, Monte Carlo (MC) simulations are a robust probabilistic technique for uncertainty quantification and model validation. They involve repeated random sampling to generate distributions of possible outcomes and assess system reliability under variability. MC methods have been vital in environmental engineering, especially when it comes to assessing the random nature of WW treatment processes. They help to explain variations in parameters (such as the variability of the influent) and provide reliable estimates of performance metrics such as removal efficiencies. By applying MC simulations (e.g., 100 iterations) to RSM-derived models, researchers can simulate diverse operational scenarios, quantify risks associated with factor interactions (such as HRT-OLR variability) and enhance the robustness of predictions for COD and turbidity removal, thereby improving decision-making in uncertain real-world conditions [19,20]. This analytical paradigm further improves with the introduction of Machine Learning (ML) algorithms, such as Random Forest (RF), to provide robustness against non-linearities, noise and overfitting through ensemble learning [21]. RF is an extension of decision trees that aggregates predictions from multiple trees trained on bootstrapped datasets. It provides feature importance rankings and achieves high predictive accuracy [22]. Accurate forecasting of membrane fouling rates using RF with an accuracy exceeding 90% has demonstrated the superiority of ML over traditional models in predicting effluent quality under variable influent conditions. This approach overcomes the limitations of standalone methods and provides predictive capabilities for dynamic, real-world scenarios [23].

Although hybrid approaches combining response surface mechanics (RSM), ML, and MC simulation have been reported for WW treatment, their application to aerobic ceramic membrane bioreactors (AeCeMBRs) remains limited. The novelty of this study lies in: (i) the integration of RSM and RF within a coherent probabilistic framework; (ii) the explicit correlation between RF predictions and MC simulation to quantify uncertainty; and (iii) the application of this framework to STW treatment using AeCeMBRs, where biological and membrane-dependent processes interact under various operational parameters including HRT, OLR, MLSS, and TMP, with a focus on its effectiveness in removing COD and turbidity, representing a significant advance in this field through the development of a robust predictive model capable of evaluating treatment performance under relatively stable filtration conditions without membrane fouling.

## 2. Materials and Methods

### 2.1. The AeCeMBR Pilot-Scale Configuration

**Figure 1:**
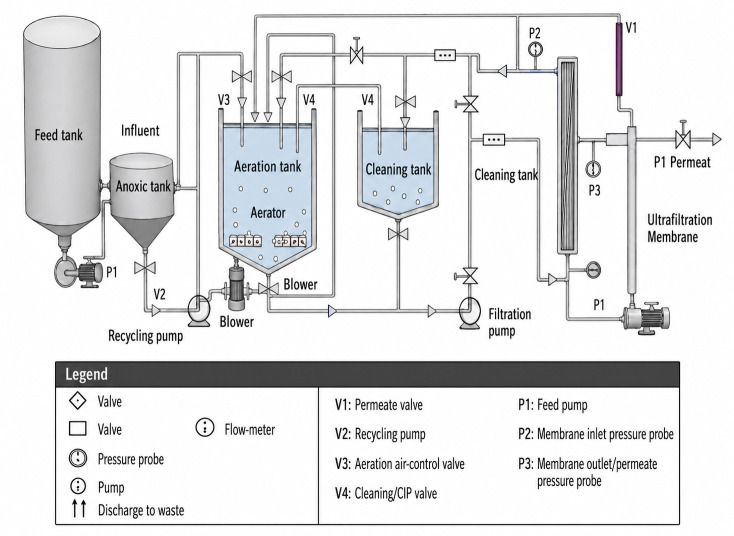
Diagram of an external membrane bioreactor system.

The treatment of STW was carried out by using a pilot-scale AeCeMBR over a period of 120 days; various combinations of four key factors were systematically tested following preliminary tests to determine the operating range, the BBD matrix, and additional performance tests. Figure 1 shows a schematic representation of the AeCeMBR pilot, which comprised a feed tank, anoxic and aerobic zones, and ceramic membrane module, with controlled aeration to support microbial activity and permeate extraction post-filtration [14]. The pilot system was constructed using COSIMI components supplied by Deltalab. Table 1 details operational parameters of the pilot-scale AeCeMBR.

### 2.2. Reagents and Membranes

The chemicals used to prepare the STW solution, including dyes and nutrient salts, were based on a formulation that was adapted from reference Suhan et al., [24]. This STW solution was treated using an AeCeMBR pilot model equipped with an UF membrane. Chemicals used in the composition of the STW are listed in Table 2, and it provides details on the specifications of the tubular ceramic membrane module, including a membrane surface area of 0.45 m^2^ and a pore size equivalent to 10–20 nm for effective solids retention.

Analytical-grade reagents were used to simulate the physicochemical parameters of typical textile effluent. The mixture was freshly prepared before each experiment. The raw STW had an initial high alkaline pH (around 9.55), which was adjusted to 7.0 ± 0.2 prior to feeding the AeCeMBR to ensure optimal biological activity. The pH was adjusted to 7.0 to standardize conditions and focus on other parameters. Table 3 summarizes the physical and chemical properties of STW.

### 2.3. Analytical Methods

All physicochemical analyses were performed in accordance with internationally accepted standard procedures to guarantee accuracy and reproducibility. The COD was determined using a dichromate reflux technique with a Hach DR2800 spectrophotometer, as per standard methods for the examination of water and WW [25]. The BOD_5_ was measured with an OxiTop WTW respirometric system in accordance with the guidelines specified by Rodier [26]. Total suspended solid (TSS) and total mixed-liquor suspended solids (MLSS) were measured following the filtration of samples through 0.45 μm pore filters, and centrifugation method. The total dissolved solids (TDS), NaCl (%), and electrical conductivity (EC) were quantified using a calibrated iNova multiparameter conductivity meter (WTW, Weilheim, Germany) with a platinum-strip conductivity cell to enable precise determination of ionic strength. A JENWAY pH-meter (Jenway, Stone, UK) was used to measure pH and temperature, turbidity was measured using a Hanna HI 98713 turbidity-meter (Hanna Instruments, Woonsocket, RI, USA), and DO was measured using an Oximeter Hanna HI 98193 (Hanna Instruments, Woonsocket, RI, USA). According to the Rodier standard [26], chloride (Cl^−^) and sulfate (SO_4_^2−^) ions were analyzed using a Jenway PFP7 flame photometer (Jenway, Stone, Staffordshire, UK), in line with the dosage method. The concentrations of nitrate (NO_3_^−^-N) were determined using a Sension MM340 ion-selective electrode (Hach, Loveland, CO, USA), in line with standard electrochemical methods. The total Kjeldahl nitrogen (TKN) was examined according to the standard Kjeldahl digestion–distillation system supplied by J.P. Selecta, S.A. (Abrera, Barcelona, Spain), following the analytical procedure described by VELP Scientifica y [27]. The concentrations of total phosphorus (Pt) and orthophosphates (PO_4_^3−^P) were determined using a colorimetric technique based on the formation of complexes with ammonium molybdate and potassium antimony tartrate in an acidic solution [26,28].

### 2.4. Response Surface Methodology Approach

The RSM is a strong statistical technique that is often used in scientific and engineering areas to improve and create models of complicated systems. RSM was created in the mid-twentieth century, and since then it has become an indispensable instrument for researchers and engineers. It is used to help them to achieve optimal outcomes with limited resources and to explore connections between several components. The fundamental concept underlies RSM, which involves constructing an approximation of a system’s response surface. Selected operating parameters (HRT, OLR, MLSS, and TMP) were identified and their ranges defined based on preliminary range-determination tests, in order to ensure realistic operating conditions for the AeCeMBR systems. The BBD was used in this study to investigate the effects and interactions of key operational parameters while reducing the number of experimental runs. It uses a three-level factorial approach. The levels are coded as −1, 0, and +1. These correspond to the minimum, central, and maximum values of each factor. A factorial combination of at least three independent factors is required in the design, arranged in partially blocked experiments. In these experiments, one factor remains constant in each block while the others vary across specified levels. Four factors were considered independently for this study: Factor 1 (HRT, h), Factor 2 (OLR, kg COD·m^−3^·d^−1^, Factor 3 (MLSS, g/L), and Factor 4 (TMP, bar)). The experimental ranges and levels for each factor are summarized in Table 4. The BBD approach allowed us to explore factor interactions systematically and cost-effectively while keeping the number of experimental runs manageable.

### 2.5. Uncertainty Analysis Using Monte Carlo Simulation

The MC simulation was used to assess the uncertainty in COD and turbidity removal under varying operating conditions using Python software 3.10.12. In the absence of detailed statistical distributions for the input parameters (HRT, OLR, MLSS, and TMP), uniform probability distributions within a range of ±10% of the experimentally studied ranges were adopted. This assumption is commonly used in environmental and engineering studies when available experimental data are limited, allowing for a limited representation of uncertainty without favoring specific values. The ±10% variance range was chosen as a conservative range reflecting the realistic fluctuations typically observed in MBR systems at an experimental scale, while avoiding excessive variability. A total of 10,000 MC simulation iterations were performed to ensure statistical convergence. Subsequently, the main probability measures, including means, standard deviations, and confidence intervals from 5% to 95%, were calculated.

### 2.6. Machine Learning (ML) Using Random Forest Coupled to MC Simulation

An ensemble ML technique, specifically an RF regression model, was developed to capture nonlinear interactions between operational parameters and enhance predictability, thereby supporting deterministic RSM analysis. It minimizes overfitting and enhances predictive ability by constructing multiple decision trees from subsets of the initial experimental data and combining their forecasts [23]. Based on 174 data points collected following a comprehensive series of tests, it includes:

• 12 data points from preliminary range-determination tests,
• 29 data points from the BBD plan (including 5 repeated at the center),
• 133 points from additional tests covering various combinations (HRT, OLR, MLSS, TMP) within the operating ranges, without any identical repetitions to the BBD.

An RF regression model was implemented using the scikit-learn library. To optimize the main parameters, hyperparameters were fine-tuned using a network search coupled with five-fold cross-validation, including the number of trees (n_estimators), maximum tree depth (max_depth), and minimum number of samples per leaf. The final model was selected based on the lowest cross-validation error. The RF algorithm implemented boot clustering and random feature selection to prevent overshoot. A trained model with 80% of data was combined with on-site MC simulations to identify robust operating zones and determine the probability of achieving the target COD and turbidity removal efficiency within the probabilistic reliability and operational window. The model’s generalizability was evaluated using a separate test dataset (20%). Model performance metrics included the coefficient of determination (R^2^) (Equation (1)), mean squared error (MSE) (Equation (2)), root mean squared error (RMSE) (Equation (3)), and mean absolute error (MAE) (Equation (4)). Plots of predicted versus experimental responses were employed to evaluate the model’s accuracy, and a feature importance analysis was conducted to rank the relative impact of HRT, OLR, MLSS, and TMP on treatment efficacy

(1) $$R^{2}=1-\frac{{\sum_{i=1}^{N}(y_{i}-\hat{y})}^{2}}{\sum_{i=1}^{N}{\left(y_{i}-\bar{y}\right)}^{2}}$$

(2) $$MSE=\frac{1}{N}{\sum_{i=1}^{N}(y_{i}-\hat{y})}^{2}$$

(3) $$RMSE=\sqrt{MSE}=\sqrt{\frac{1}{N}{\sum_{i=1}^{N}(y_{i}-\hat{y})}^{2}}$$

(4) $$MAE=\frac{1}{N}\sum_{i=1}^{N}\left|y_{i}-\left.\hat{y}\right|\right.$$

where the ith sample from the target variable is denoted by y_i_, the predicted outcome is represented by $\hat{y}$, and the mean of the collected data is signified by $\bar{y}$ [29].

## 3. Results and Discussion

### 3.1. RSM Modeling and Statistical Evaluation of AeCeMBR Performance

Assessing the surface of response allows researchers to predict the optimal settings for factors, identify variables with significant effects, and explore the system’s behavior within the scope of the experiment [30]. A BBD was used to systematically investigate the effects of four key operational parameters on the removal efficiencies of COD and turbidity (Y1 and Y2) in a pilot-scale AeCeMBR treating STW. The parameters investigated were hydraulic retention time (HRT, A), organic loading rate (OLR, B), mixed liquor suspended solids (MLSS, C) and transmembrane pressure (TMP, D). The RSM employed a BBD for 29 experimental runs. These included five centre points. These were used to estimate pure error and assess model curvature. This ensured adequate statistical reliability. At the same time, it minimized the number of experiments. The BBD is particularly suitable for quadratic modelling because, unlike full three-level factorial designs, it positions experimental points on a spherical region and avoids the vertices of the cubic design space [31,32]. Table 5 shows the experimental design matrix and the matching responses. COD removal efficiencies ranged from 94.7% to 97.7%, while turbidity removal varied from 95.0% to 98.1%. This indicates that treatment performance depends strongly on operating conditions. The improvement in COD and turbidity removal is all the more significant as the HRT is maintained at a particular level [33]. When HRT is increased from 6 to 15 h, there is a noticeable improvement in both COD and turbidity removal; COD removal rises from approximatively 95% to values above 97%. This behavior is a reflection of enhanced biodegradation of textile organics due to an extended contact time between microorganisms and recalcitrant compounds [34]. Elevated MLSS levels (10 g/L) further improved performance, yielding COD removals above 96.6% and turbidity removals greater than 97.1%. This is due to increased biomass concentration and improved solids retention. However, when combined with lower HRT or MLSS, higher OLR values (3 kg COD·m^−3^·d^−1^) resulted in a slight decrease in efficiency, suggesting substrate overloading and increased fouling propensity. TMP exhibited a nonlinear effect, with intermediate values (0.65–1.2 bar) favoring optimal removal, while lower TMP resulted in limitations to treatment efficiency and permeate driving force [35]. Minimal variability in the center-point runs confirmed low pure error and good experimental reproducibility [36]. In this study, the TMP was maintained within a range where fouling remained moderate (0.1–1.2 bar) in order to isolate the effect of operating parameters on permeate quality.

Table 6 presents the ANOVA results for the quadratic RSM models developed for COD and turbidity removal. A high F-value of 25.34 with *p* < 0.0001 is exhibited by the COD model, while an F-value of 23.91 with *p* < 0.0001 is shown by the turbidity model, confirming that both models are highly significant. The models account for over 95% of the experimental variability, as evidenced by the high coefficients of determination (R^2^ = 0.96 and 0.95 for COD and 0.95 for turbidity). The lack-of-fit values for COD (0.17) and turbidity (0.18) were non-significant, and the pure error was low (0.02), demonstrating that the quadratic equations adequately describe the system behavior without unmodeled curvature. These statistical indicators confirm the suitability of RSM for capturing the nonlinear interactions governing AeCeMBR performance under STW treatment parameters. In accordance with the statistically significant results of the ANOVA analysis, the experimental data were modelled using second-order polynomial equations to describe the removal of COD and turbidity as a function of the four operational variables (HRT, OLR, MLSS, and TMP). The resulting regression equations are expressed as follows:

(5) Y1 = 95.77 + 0.8934A + 0.2927B + 0.4891C + 0.2870D + 0.1461AB + 0.0329AC − 0.3415AD − 0.0502BC + 0.0468BD − 0.3561CD + 0.0199A^2^ + 0.1152B^2^ + 0.2161C^2^ + 0.3072D^2^

(6) Y2 = 96.12 + 0.9266A + 0.2882B + 0.5045C + 0.2933D + 0.1297AB − 0.0006AC − 0.3230AD − 0.0760BC + 0.0472BD − 0.3550CD + 0.0305A^2^ + 0.1800B^2^ + 0.2396C^2^ + 0.3750D^2^

According to the regression equations, HRT (A) and MLSS (C) exhibit the strongest positive linear effects on both COD and turbidity removal, reflecting enhanced solids retention and increased biological activity at higher levels [37]. The positive quadratic terms, particularly for TMP (D^2^), indicate diminishing returns at extreme operating conditions, primarily due to intensified membrane fouling and cake layer compression, which lead to a significant reduction in porosity and an increase in filtration resistance. Negative interaction terms such as AD and CD reveal antagonistic effects, suggesting that elevated TMP combined with high HRT or MLSS can adversely affect system performance by restricting permeate transport [37]. These regression models provide the mathematical basis for the subsequent response surface analysis and multi-response process optimization [17].

**Figure 2:**
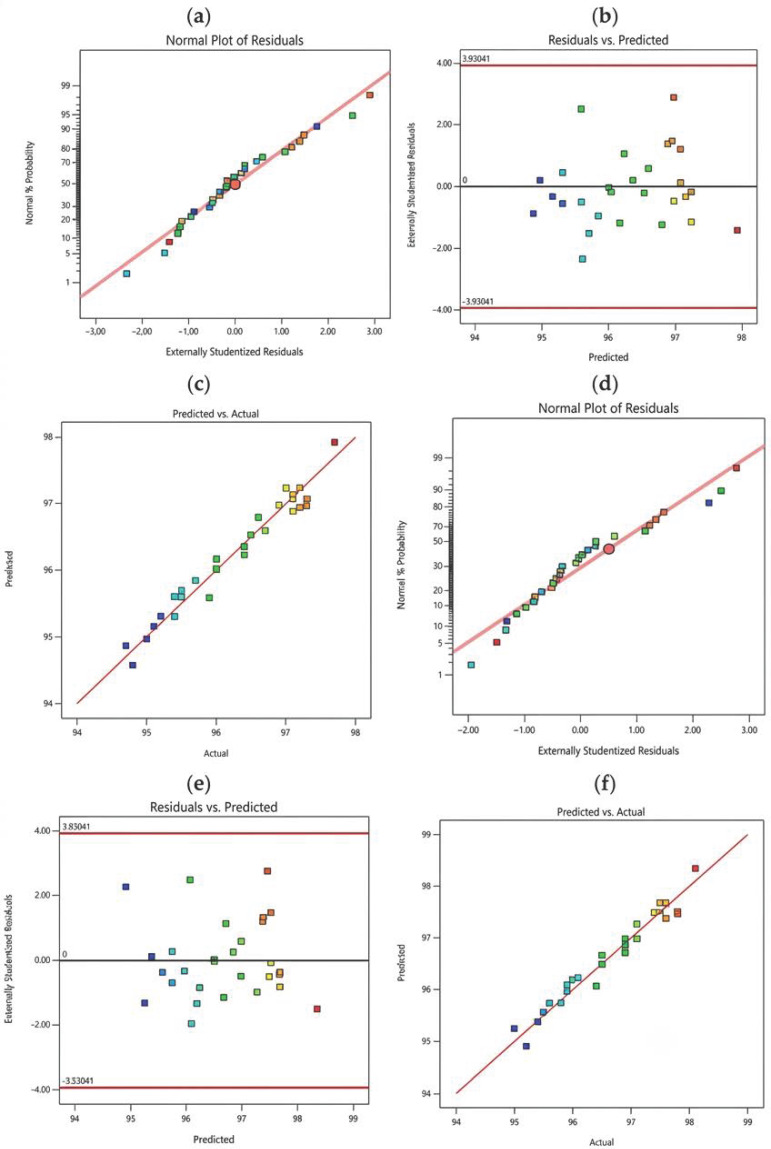
Plots of normal probability of residuals (**a**–**d**) residuals vs. predicted values (**b**–**e**) and predicted vs. actual values (**c**–**f**) for COD and Turbidity removal.

Figure 2 presents the diagnostic plots that comprehensively validate the adequacy and statistical integrity of the RSM model. The normal probability plots of residuals (Figure 2a,d) show that the externally studentized residuals are approximately linearly distributed. This confirms that the normality assumption is satisfied. It also confirms that the errors are independently distributed. When the residuals are analyzed against the predicted values (Figure 2b,e), a random scatter of data points within the experimental limits is observed, with no signs of funneling or patterns, which suggests constant variance and the absence of systematic bias in the model. The plots of predicted versus actual values (Figure 2c,f) demonstrate a strong correlation, with the data points closely grouped around a 45° line. The minimal deviation between experimental and predicted values confirms the high predictive power and robustness of the quadratic models for COD and turbidity removal. Therefore, these diagnostic results justify the use of the developed models for process optimization and subsequent surface analysis.

**Figure 3:**
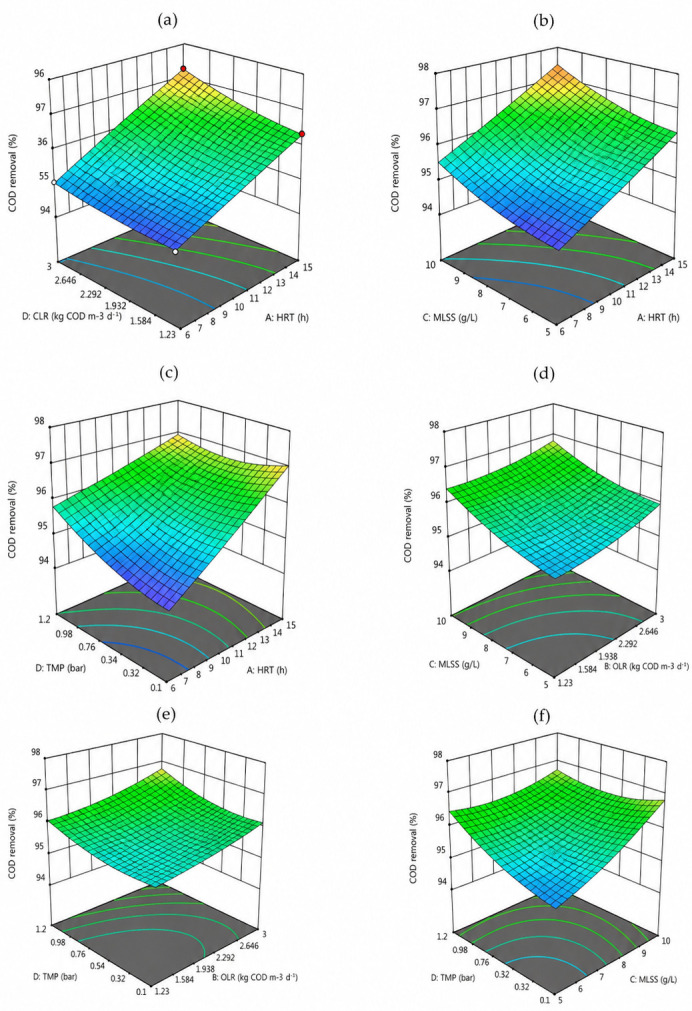
3-D Response surface plots displaying the interactive effects of OLR and HRT (**a**), MLSS (**b**) TMP and HRT (**c**), MLSS and OLR (**d**), TMP and OLR (**e**), TMP and MLSS (**f**) on the response (COD removal, Y1), where A = HRT, B = OLR, C = MLSS, D = TMP.

**Figure 4:**
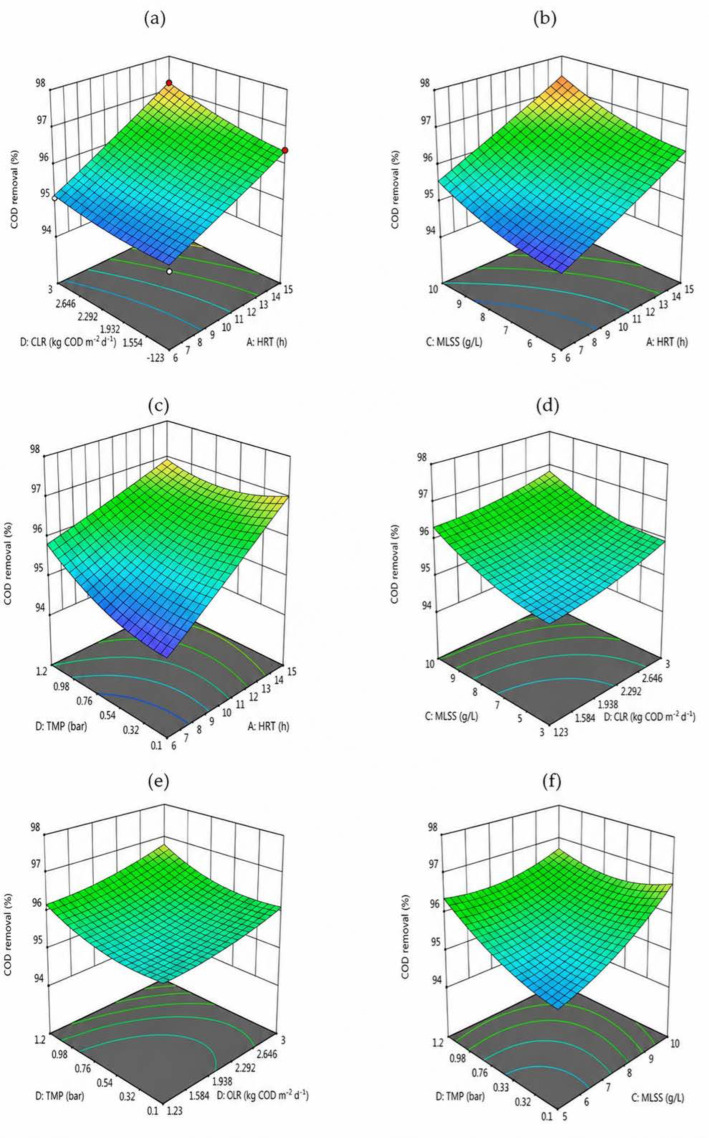
3-D Response surface plots displaying the interactive effects of OLR and HRT (**a**), MLSS (**b**) TMP and HRT (**c**), MLSS and OLR (**d**), TMP and OLR (**e**), TMP and MLSS (**f**) on the response (Turbidity removal, Y2), where A = HRT, B = OLR, C = MLSS, D = TMP.

The interactive effects of the operating parameters on COD and turbidity removal are described by the three-dimensional response surface plots shown in Figure 3 and Figure 4, respectively. The curved convex surfaces and elliptical contour projections confirm that both responses follow a quadratic behavior, which indicates statistically significant interactions between factors. Figure 3 demonstrates how the interaction between HRT and OLR reveals a convex surface, with maximum COD removal (>97%) achieved at high HRT and moderate OLR [38]. In this case, extending HRT promotes biodegradation without substrate inhibition. The HRT–MLSS interaction exhibits a clear synergistic effect, with COD removal exceeding 96% for MLSS values of 7–9 g/L and HRT values greater than 8 h, reflecting enhanced biomass-driven degradation. Conversely, TMP-related interactions exhibit antagonistic trends at higher TMPs, attributable to cake layer compression and reduced permeate transport [39].

Figure 4 shows that turbidity removal is more strongly influenced by TMP and MLSS, as indicated by steeper response curves. This is consistent with the dominance of physical separation mechanisms. Optimal turbidity removal (>97%) occurs at intermediate TMP values, where maximized particulate retention is achieved while avoiding excessive fouling. Overall, these response surfaces clearly highlight the nonlinear trade-offs between biological treatment efficiency and membrane separation performance [40], providing valuable insight into the optimal operating window of the AeCeMBR system.

The results of multi-response optimization using the desirability function approach are summarized in Table 7. The optimal operating conditions identified were HRT = 10.39 h, OLR = 1.91 kg COD·m^−3^·d^−1^, MLSS = 7.66 g/L and TMP = 1.08 bar. While experimental validation yielded higher removal efficiencies for COD (97.7%) and turbidity (98.1%), the model predicted values of 96.1% and 96.5%, respectively, with relative deviations of less than 2%. This close agreement confirms the reliability of the RSM models’ predictions and demonstrates that high treatment efficiencies can be achieved under moderate operating conditions without excessive energy demand. The RSM findings of this study demonstrate the superior performance of the AeCeMBR system for treating textile WW. Although their statistical performance is comparable, using four polynomial regression models to predict efficiency, their removal values are lower than the 97.7% COD and 98.1% turbidity removal values achieved in the present study. This is indicative of the enhanced biodegradation and solids retention that is required for textile WW containing more recalcitrant compounds. The higher performance achieved in this work is explained by the specific optimal parameters identified for the complex textile WW matrix, in contrast to the work performed by A. V. Sonawane and Z. V. P. Murthy [41] where HRT of 6 h and sludge retention time of 40 d, was used to control fouling.

### 3.2. Improving the Performance of AeCeMBR Using Monte Carlo Simulation and Random Forest

Table 8 presents the statistical outcomes of the MC simulations. These variables quantify the uncertainty and operational risk for both COD and turbidity removal under ±10% input variability. The mean value of the MC analysis for COD removal was 96.71%, with a low standard deviation of ±0.55%. This resulted in a narrow 5–95% confidence interval (95.89–97.63%). The target COD removal of ≥97% was 68%, indicating a high likelihood of compliance under realistic operating fluctuations. In contrast, turbidity removal exhibited a lower mean (84.96%) and a higher standard deviation (±1.75%), a wider confidence interval (82.04–87.77%) and a reduced probability (52%) of meeting the ≥87% target. This difference shows that turbidity removal is more sensitive to stochastic fouling behavior [42]. As a result, Table 8 provides crucial risk-based insight that is difficult to obtain using only deterministic RSM or ML predictions.

**Figure 5:**
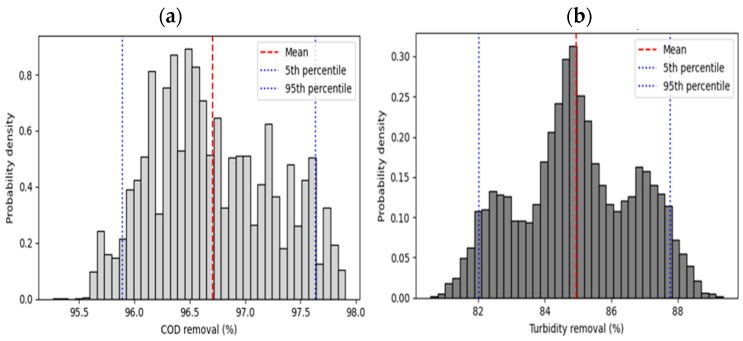
Monte Carlo simulation—COD removal (**a**) and Turbidity removal (**b**).

Figure 5a shows the MC probability distribution for COD removal, which was generated from 10,000 iterations under ±10% uncertainty in operating parameters. The histogram shows a near-symmetric, bell-shaped distribution, which is centered at a mean COD removal of 96.71%, with a low standard deviation of 0.55%, which indicates a high degree of system stability. The narrow 5–95% confidence interval (95.89–97.63%) indicates minimal sensitivity of COD removal to moderate variations in HRT, OLR, MLSS, and TMP. The pronounced peak around 96.5–97.0% signifies effective biological degradation, while the short tails represent infrequent unfavorable conditions such as transient organic overload or diminished biomass activity. Figure 5a demonstrates that COD removal in the AeCeMBR is highly resilient and that deterministic optimization outcomes are supported by strong probabilistic confidence. Figure 5b shows the MC distribution for turbidity removal, which is wider than the COD distribution. With a larger confidence interval of 5–95% from 82.04 to 87.77% and a higher standard deviation of 1.75%, the average turbidity removal is 84.96%. Particle size variation, cake layer restructuring, and the random nature of membrane fouling contribute to increased dispersion [43]. These factors add a higher degree of uncertainty to physical filtration processes. The distribution shows slight asymmetry, indicating that favorable hydrodynamic conditions can enhance turbidity removal, while adverse fouling conditions may reduce performance [44]. It is clear from Figure 5b, that turbidity removal is inherently more volatile than COD removal, meaning that it requires a probabilistic rather than deterministic assessment (RSM). MC simulation was applied by V. Ganthavee et al., [45] to quantify uncertainty in dye removal efficiency within a three-dimensional electrochemical WW treatment system, with uncertainty ranges of approximately 3–6% under operational variability being reported. This is similar to the results of the present study, which showed that physical removal mechanisms are more variable than bulk organic degradation. By contrast, the AeCeMBR system examined here exhibited a more confined uncertainty range for COD removal (Std = 0.55%) and a more extensive distribution for turbidity removal (Std = 1.75%), underscoring the stabilizing effect of biological oxidation and the inherently random nature of membrane filtration in textile WW treatment.

**Figure 6:**
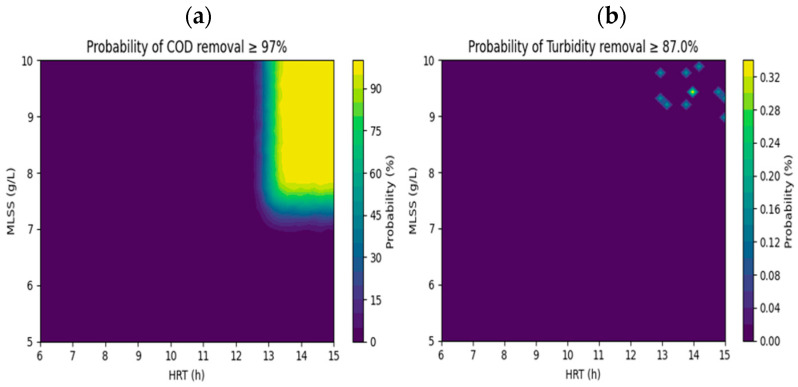
Probability of COD removal (**a**) and Turbidity removal (**b**).

The probability of achieving target removal efficiencies (≥97% COD and ≥87% turbidity) was estimated using fixed operating conditions (OLR = 2.18 kg COD·m^−3^·d^−1^ and TMP = 0.65 bar). The findings were presented using probability diagrams and cumulative distribution functions, which supplied a risk-based evaluation of AeCeMBR operational reliability. Figure 6 illustrates the spatial probability distribution for achieving COD removal ≥97%. A distinct region of high probability (>70–90%) emerges at an HRT ≥11 h and an MLSS of ≥7.5 g/L, indicating a robust operational window in which biological degradation is maximized. Outside this region, the probability declines sharply, particularly at a low HRT (<9 h), suggesting that there is insufficient contact time for microbial oxidation. Figure 6a translates statistical uncertainty into practical advice, enabling operators to choose operating conditions that keep high COD removal with measured certainty rather than relying only on point predictions. Figure 6b shows the probability of achieving turbidity removal of at least 87% under the same fixed OLR and TMP conditions. The high-probability region is significantly smaller than that of COD, with maximum probabilities of only ≈50–55%, concentrated around HRT ≈9–11 h and higher MLSS values. This suggests that turbidity removal is more susceptible to fouling dynamics and operational disruptions. High turbidity removal cannot be assured with the same level of confidence as COD removal, even in ideal circumstances. This highlights the significance of proactive fouling management techniques and cautious operating margins in the treatment of textile WW.

The predictive performance of the RF model combined with MC simulation provides a robust, uncertainty-aware decision-making tool for optimizing AeCeMBR under realistic operational fluctuations. RF regression with MC simulation, developed for COD and turbidity removal under optimized AeCeMBR operating conditions, is illustrated by Table 9. The RF-MC model for COD removal achieved a high coefficient of determination (R^2^ = 0.926). This indicates that over 92% of the experimental variability was accurately captured. The RF model demonstrated superior performance in terms of turbidity removal, indicating exceptional predictive accuracy. The suitability of RF for engineering-scale prediction is confirmed by the consistently low MAE and RMSE values for both responses (<0.25%), particularly in systems characterized by nonlinear biological kinetics and membrane filtration behavior [23]. Higher R^2^ and lower errors were obtained for turbidity. This indicates that physical separation processes are more consistently predictable. This is in contrast to biologically mediated COD removal. This is inherently more sensitive to microbial dynamics and organic loading fluctuations.

**Figure 7:**
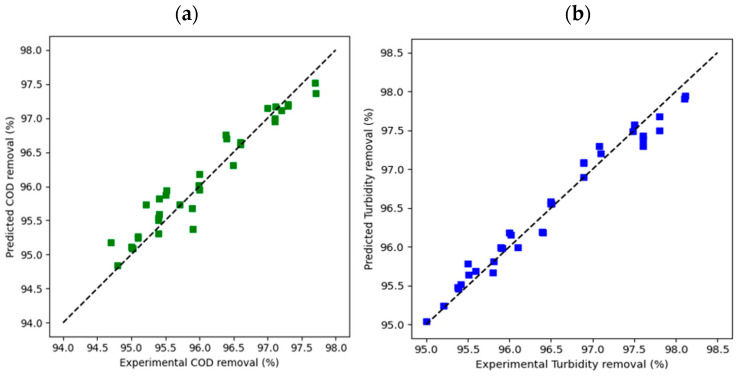
Experimental vs. predicted of COD removal (**a**) and Turbidity removal (**b**).

The experimental and RF-MC algorithm in Figure 7a displays the predicted values of COD removal, thereby visually verifying the model’s performance. The COD removal plot shows that the data points cluster tightly reference line across the entire removal range (94–98%), indicating excellent agreement between measured and predicted values. The limited dispersion and absence of systematic bias demonstrate that the RF-MC model generalizes well and is not subject to overfitting, even at removal efficiencies exceeding 97%. Reproducing rare optimal operating conditions, reflected by slight dispersion in the upper removal range, is inherently challenging even with data augmentation; however, the deviations remain below ±0.5%, confirming the high predictive accuracy of the model. Figure 7b illustrates the experimental versus RF-MC models for predicted values of turbidity removal. This demonstrates excellent predictive capability of the RF-MC models. The data points are closely clustered around the reference line for the whole turbidity removal range (around 95–99.5%), showing a strong agreement between measured and predicted values. The minimal scatter and the lack of systematic deviation confirm that the RF-MC model generalizes well and maintains high accuracy under varying operating conditions.

A comparative analysis of recent ML studies has confirmed the adequacy and robustness of RF model for performance prediction in AeCeMBR. N. Nadeem et al., [46] integrate Gaussian Process Regression (GPR) with MC simulations to improve the predictive accuracy in WW treatment regarding methylene blue degradation in photocatalytic WW treatment, achieving a relative percentage absolute error of 0.926 and moderate pair-wise correlations of around 0.51, thus demonstrating GPR’s effectiveness in smooth, low-noise nonlinear systems. In comparison, the current RF-MC model obtained comparable coefficients of determination (R^2^ = 0.926 for COD and 0.971 for turbidity) with reduced prediction errors (RMSE < 0.24), even though it operated in a complex MBR environment characterized by fouling dynamics, biomass variability, and hydraulic-shear interactions. GPR relies on kernel assumptions. The RF captures threshold effects and regime shifts effectively. They are inherent to membrane systems. The RF framework offers explicit feature importance rankings, providing operational insights that are often lacking in GPR-based approaches. The results show that RF combined with MC simulation is particularly well suited for MBR systems under variable operating conditions. The significance analysis of the features revealed that HRT and MLSS were the variables with the greatest impact on COD removal, confirming the dominant role of biodegradation, while TMP showed a greater impact on turbidity removal, reflecting membrane-driven separation mechanisms.

### 3.3. Comparative Evaluation of RSM and RF–MC Approaches

Using both RSM and the RF–MC approach together lets us fully test how well a model works under different conditions. Overall, the RF–MC framework was better at making predictions than the RSM, especially when dealing with nonlinear relationships and changes in the process. The RF–MC model made predictions that were more accurate and stable, with less uncertainty and fewer differences between what was predicted and what was actually observed.

On the other hand, the RSM approach showed more spread in prediction errors and less consistency, especially when the operating conditions were complicated. The enhanced performance of RF–MC is due to its capacity to capture nonlinear interactions and incorporate uncertainty via MC simulation. Table 10 shows the best operating conditions that come from the RF–MC approach.

Similar results have been reported in previous studies. In a previous study [21], RSM was compared to a Support Vector Machine–Monte Carlo (SVM–MC) method, and it was found that machine learning-based models made predictions more accurately and reliably. In the current study, RSM yielded analytically interpretable optimal conditions, whereas the RF–MC framework delivered more dependable predictions with reduced uncertainty ranges, rendering it more appropriate for intricate AeCeMBR systems.

Based on the RF-MC framework, the optimal operating conditions shown in Table 10 are when both COD removal and turbidity are optimized simultaneously.

These results indicate that the identified conditions provide a balanced optimization between the two performance indicators while maintaining low levels of uncertainty. COD removal is primarily driven by biodegradation processes and is less sensitive to moderate operational fluctuations. In contrast, turbidity removal is mainly influenced by membrane filtration mechanisms and is more susceptible to membrane fouling and hydrodynamic changes. This explains the slightly wider uncertainty range observed for turbidity compared to COD.

## 4. Conclusions

This study presented and evaluated the RSM, RF and MC simulation framework for the optimization and evaluation of an AeCeMBR treating textile industry WW. The modelling methodology allows for systematic optimization of process, precise modelling of non-linear system behavior and rigorous quantification of operational uncertainty. This addresses the main limitations of conventional single-method approaches. The results show that biological performance in the AeCeMBR system is very robust under optimized conditions. However, physical separation processes are more sensitive to operational variability. This underscores the need to account for uncertainty consciously. The proposed framework is a reliable decision-support tool for process design, control, and scale-up. This is achieved by coupling ML using RF regression prediction and probabilistic risk analysis. With the potential to be readily adapted to other membrane-based treatment systems dedicated to high variability of influent, this method offers a workable solution for the efficient treatment of complex textile WW. This study demonstrates the potential of an integrative framework that combines the RSM, RF, and MC models to improve the performance of the AeCeMBR system under conditions of uncertainty. However, there are still some limitations related to the lack of pollution modeling, the use of industrial wastewater, and the relatively small dataset. In order to improve the model’s robustness and generalizability, the next step of research will focus on incorporating real wastewater conditions, pollution dynamics, and larger or longer-term datasets, and testing the pH range (7–10) using a biomass tailored for alkalophilic organisms, or employing a physical-chemical pretreatment step.

## Figures and Tables

**Table 1:** Operating conditions of the pilot-scale AeCeMBR.

AeCeMBR Operating Conditions	pH	6.5–8
	Temperature	23 ± 4 °C
	HRT	6–15 h
	Aeration rate	700–1000 NL/h
	Dissolved Oxygen (DO)	2–4 mg/L
	Permeate flux	11.1–51.1 L/m^2^h
	TMP	0.1–1.2 bar
	MLSS	5–10 g/L
	OLR	1.23–3 kg COD·m^−3^·d^−1^

**Table 2:** UF membrane characteristics of the pilot-scale AeCeMBR.

Chemicals Used	Materials	Concentration
	Starch	1000 mg/L
	Acetic acid	0.20 mL/L
	Dye (Methylene Blue)	251 mg/L
	Caustic soda	512.5 mg/L
	Sodium carbonate	501.5 mg/L
	Sulfuric acid	0.301 mg/L
	Detergents	128.5 mg/L
	Sodium chloride	3076.5 mg/L
**UF Membrane**	Material & Module	Ceramic & Tubular type P10
	Cut off	15 kD/10 to 20 nm
	Membrane area	0.45 m^2^
	Membrane length	1.178 m
	Diameter of the channels	6 mm

**Table 3:** Physicochemical parameters of STW.

Physicochemical Parameters	Parameters	Concentration
	Turbidity	74.9 NTU
	TDS	2.5 g/L
	Electrical conductivity	4.86 mS/cm
	pH	9.55
	TS	5.30 g/L
	TSS	1.9 g/L
	COD	520 mg/L
	BOD	176 mg/L
	PO_4_^3−^-P	2.53 mg/L
	Pt	5.2 mg/L
	NO_3_^−^-N	20.05 mg/L
	SO_4_^2−^	104 mg/L
	Cl^−^	2.33 mg/L
	Color	Blue
	NaCl	9.4%

**Table 4:** Experimental ranges used in the BBD.

Independent Factors	Units	Codes	Levels		
			−1	0	+1
HRT	h	A	6	11.16	15
OLR	kg COD·m^−3^·d^−1^	B	1.23	2.20	3
MLSS	g/L	C	5	7.30	10
TMP	bar	D	0.1	0.628	1.2

**Table 5:** Experimental values of the BBD and responses.

Run	A, HRT (h)	B, OLR (Kg COD·m^−3^·d^−1^)	C, MLSS (g/L)	D, TMP (bar)	Y1, COD Removal (%)	Y2, Turbidity Removal (%)
1	6	1.23	7.5	0.65	94.7	95
2	15	1.23	7.5	0.65	96.4	96.9
3	6	3	7.5	0.65	95.1	95.5
4	15	3	7.5	0.65	97.2	97.6
5	10.97	2.18	5	0.631	95.5	95.9
6	10.97	3	5	0.631	96	96.5
7	10.97	2.18	10	0.631	96.7	97.1
8	10.97	3	10	0.631	97.2	97.6
9	10.97	2.18	5	0.1	95.2	95.6
10	10.97	2.18	5	0.1	95.4	95.8
11	10.97	2.18	10	1.2	96.6	97.1
12	10.97	2.18	5	1.2	96.5	96.9
13	6	2.18	10	0.1	95.4	95.9
14	15	2.18	7.5	0.1	97.1	97.5
15	6	2.18	7.5	1.2	95.7	96.1
16	15	2.18	7.5	1.2	97.3	97.8
17	10.97	1.23	7.3	0.1	95.5	96
18	10.97	3	7.3	0.1	96.4	96.9
19	10.97	1.23	7.3	1.2	96	96.5
20	10.97	3	7.3	1.2	97.1	97.6
21	6	1.23	5	0.65	94.8	95.2
22	15	1.23	5	0.65	96	96.5
23	6	3	5	0.65	95	95.4
24	15	3	5	0.65	96.9	97.4
25	6	1.23	10	0.65	95.9	96.4
26	15	1.23	10	0.65	97.1	97.6
27	15	3	10	0.65	97.7	98.1
28	15	3	7.5	0.65	97	97.5
29	15	2.18	7.5	0.1	97.3	97.8

**Table 6:** ANOVA results of the quadratic model for COD and Turbidity removal.

Response	Degree of Freedom	Sum of Squares	Mean Squares	F-Value	*p*-Value	Significance	R^2^	Lack of Fit	Pure Error
Y1	14	20.55	1.47	25.34	˂0.0001	++	0.96	0.17	0.02
Y2	14	21.55	1.54	23.91	˂0.0001	++	0.95	0.18	0.02

The “++” symbols in the Significance column mean a significant positive effect/significance (corresponding to the *p*-value < 0.0001).

**Table 7:** Response optimization by BBD.

HRT A	OLR B	MLSS C	TMP D	COD Removal Y1		Turbidity Removal Y2	
				Experimental	Predicted	Experimental	Predicted
10.388	1.908	7.664	1.076	97.7	96.1	98.1	96.5

**Table 8:** Monte Carlo Simulation Summary.

Response	Mean (%)	Std	5–95% Interval	Target Probability (%)
COD Removal	96.71	0.55	95.89–97.63	68 (≥97%)
Turbidity Removal	84.96	1.75	82.04–87.77	52 (≥87%)

**Table 9:** RF Model Performance Metrics.

Response	R^2^	MSE	MAE	RMSE
COD removal	0.926	0.057	0.187	0.239
Turbidity removal	0.971	0.023	0.131	0.153

**Table 10:** Optimal operating conditions by RF-Monte Carlo algorithm.

Parameter	Value
HRT (h)	15
OLR (kg COD·m^−3^·d^−1^)	1.23
MLSS (g/L)	10
TMP (bar)	0.65
Predicted COD removal (%)	97.6
Observed COD removal (%)	97.3
IC 95% COD removal (%)	96.7–98.3
Predicted turbidity removal (%)	98
Observed turbidity removal (%)	97.8
IC 95% turbidity removal (%)	97.1–98.7

## Data Availability

Datasets generated during the study are available from the corresponding author upon reasonable request. No data was used for the research described in the article.

## Abbreviations

AeCeMBR	Ceramic aerobic membrane bioreactor
BBD	Box–Behnken design
BOD	Biochemical oxygen demand
COD	Chemical oxygen demand
DO	Dissolved Oxygen
GPR	Gaussian Process Regression
HRT	Hydraulic Retention Time
MAE	Mean absolute error MLSS
MBR	Membrane Bioreactor
MC	Monte Carlo
MLSS	Mixed Liquor Suspended Solids
ML	Machine Learning
MSE	Mean squared error
OLR	Organic Loading Rate
R^2^	Coefficient of determination
RF	Random forest
RF-MC	Random forest-Monte Carlo
RMSE	Root mean squared error
RSM	Response surface methodology
STW	Synthetic textile wastewater
TMP	Transmembrane Pressure
TSS	Total Suspended solids
UF	Ultrafiltration
WW	Wastewater
